# Biological impact of geometric uncertainties: what margin is needed for intra-hepatic tumors?

**DOI:** 10.1186/1748-717X-5-48

**Published:** 2010-06-03

**Authors:** Hsiang-Chi Kuo, Wen-Shan Liu, Andrew Wu, Dennis Mah, Keh-Shih Chuang, Linda Hong, Ravi Yaparpalvi, Chandan Guha, Shalom Kalnicki

**Affiliations:** 1 Department of Radiation Oncology, Montefiore Medical Center, USA; 2 Department Biomedical Engineering and Environmental Sciences, National Tsing Hua University, Taiwan; 3 Department of Radiation Oncology, University Hospital of Chung-Shan Medical University, Taiwan; 4 Department of Radiologic Sciences, Thomas Jefferson University, USA

## Abstract

**Background:**

To evaluate and compare the biological impact on different proposed margin recipes for the same geometric uncertainties for intra-hepatic tumors with different tumor cell types or clinical stages.

**Method:**

Three different margin recipes based on tumor motion were applied to sixteen IMRT plans with a total of twenty two intra-hepatic tumors. One recipe used the full amplitude of motion measured from patients to generate margins. A second used 70% of the full amplitude of motion, while the third had no margin for motion. The biological effects of geometric uncertainty in these three situations were evaluated with Equivalent Uniform Doses (EUD) for various survival fractions at 2 Gy (SF_2_).

**Results:**

There was no significant difference in the biological impact between the full motion margin and the 70% motion margin. Also, there was no significant difference between different tumor cell types. When the margin for motion was eliminated, the difference of the biological impact was significant among different cell types due to geometric uncertainties. Elimination of the motion margin requires dose escalation to compensate for the biological dose reduction due to the geometric misses during treatment.

**Conclusions:**

Both patient-based margins of full motion and of 70% motion are sufficient to prevent serious dosimetric error. Clinical implementation of margin reduction should consider the tumor sensitivity to radiation.

## Background

Primary hepatocellular carcinoma (HCC) and liver metastases are common in East Asia and Africa. The volume of liver cancer patients in the United States increases each year [1]. Due to the poor tolerance of the whole liver to radiation, radiation therapy (RT) has conventionally played a very limited role in treating liver cancer. Recently, advanced RT techniques (3D conformal & stereotactic radiotherapy) have been applied to unresectable focal intrahepatic cancer to improve the local control rate without serious radiation-induced liver disease (RILD) [2,3]. Michigan's group [2] has showed that HCC treatment with RT is promising. In particular, the response rate, measured by the shrinkage of the tumor volume, could be as high as 90%. In 2002, HC Park et al [4] found the response rates of HCC were 29.2%, 68.6%, and 77.1% for doses 40 Gy, 40-50 Gy, and 50 Gy, respectively (corresponding to a BED of 47.2 Gy, 47.2-59 Gy, and 59 Gy, respectively). Another group [5] found that the response rates were 46.7% in biological equivalent dose (BED) <50 Gy and 72.8% in BED > 50 Gy. For the treatment of HCC with portal vein thrombosis (PVT), two other groups [6,7] also showed a dose dependence of the local tumor response. These clinical results [2-6] &[7] reveal that intra-hepatic tumor radiation response is dose dependent regardless of the presence of PVT.

Another potential biologic marker of local recurrence after radiotherapy is the intrinsic tumor radiosensitivity. Using SF_2 _(surviving fraction of tumor cell colony at 2 Gy) as an end point for intrinsic radiosensitivity, some clinical studies have evaluated the correlation of SF_2 _with clinical stage as an independent prognostic factor of local tumor control [8-10]. These studies demonstrated a close association of SF_2 _with recurrence of cervix, and head and neck tumors, but not glioblastomas. The mechanism of radiosensitivity of hepatocarcinoma cells after radiotherapy is not well understood. However, laboratory studies [11] have confirmed that SF_2 _was significantly correlated with the hepatic carcinoma cell radiosensitivity. Since radiosensitivity is an important factor influencing the prognosis of radiotherapy treatment, it is important to consider the radiation response for different clinical stages and the tumor cell types in the treatment of intra-hepatic tumors.

An intra-hepatic tumor is a lesion situated within the abdomen, which has great geometric uncertainty due to respiratory motion (1~2.5 cm) [12] and daily setup variations (0.5~1 cm). These uncertainties may affect the radiotherapy treatment outcome especially for Intensity Modulated Radiotherapy (IMRT) delivery. The effects of organ motion on dose delivery by dynamic multi-leaf collimators (DMLC) have been studied extensively [13-15]. These results showed that the interplay between MLC leaf motion and organ motion did not influence the expected dose to the moving organ in highly fractionated IMRT delivery (approximately thirty fractions). They also found that a one cm motion margin is clinically acceptable in terms of fluence distortion and CTV (clinical target volume) coverage. Investigations of the influence of setup uncertainty on target coverage have focused on less mobile targets such as on head and neck and prostate cancer [16,17]. Recently Balter [18] evaluated the setup uncertainties on treatment plans for focal liver tumors and found the change of the effective normal liver volume difference was within 3%.

Geometric uncertainties are traditionally overcome by adding adequate margin to CTV to ensure target dose coverage and normal tissue sparing. ICRU (International Commission on Radiation Units and Measurements) report 62 [19] introduced the concept of an internal margin (IM) to take into account variations in size, shape, and position of the CTV in reference to the patient's anatomical reference points and also the concept of a setup margin (SM) to take into account all uncertainties in patient-beam positioning in reference to the treatment machine coordinate system. Report 62 suggests, instead of adding the internal margin and the setup margin linearly, a compromise has to be sought. The majority of the margin schemes are aimed at maintaining a minimum dose (e.g. 95% of the prescribed dose) to the CTV for a majority (e. g. 90%) of a patient population or a group of test plans [20,21]. For liver, the uncertainty attributed to respiratory motion is large compared to other setup errors and should be considered separately. Mckenzie et al [22] proposed a full respiratory motion amplitude (*A*) be added on top of other errors. Ten Haken et al [23] proposed the elimination of the respiratory margin while escalating the dose to an amount with the same normal tissue complication probability (NTCP) of normal liver. Van Herk et al [21] proposed a 0.7 *A *margin for motion amplitudes larger than one cm. These studies compared the geometrical impact for fractionated treatment with or without biological model. Molinelli et al [24] compared different margin protocols with 0 mm, 5 mm, and 10 mm in either radial or cranial-caudal directions for SBRT treatment of liver with single tumor type of SF_2 _= 0.5.

Table 1 summarizes the margin recopies and purposes of these studies. None of these studies correlate the size of margin with different clinical stage or the cell types of different radiosensitivity. Since the clinical stage and the radiosensitivity of different cell types are important prognostic factors of the tumor response and tumor control, we hypothesize that the margin defined for intrahepatic tumors should also consider variations in radiosensitivity which would depend upon different tumor cell types and different clinical stages. Here, different proposed margin schemes were compared for the dose smearing results on the targets with the same geometric uncertainties. EUD is an approach which calculates a uniform dose value from a non-uniform dose distribution that would result in the same biological effects (the survival of the same number of clonogens) in both. The non-uniform dose distributions after the dose smearing were converted into EUD at different SF_2 _values to evaluate the biological impact of geometric uncertainties. After correlating SF_2 _of the HCC tumor cell with different cell types and HCC stage, the biological impact of the geometric uncertainty was taken into account to guide the clinical decision of creating margins for different stages of intra-hepatic tumors.

**Table 1:** Summary of motion margin recipes and the study designs.

Author	Recipe	Biological model	Purpose
Haken et al. 1997	0A	NTCP (Lyman model) TD_50 _= 45 Gy, m = 0.15, m = 0.69; TCP (Simple logistic function) D_50 _= 60 Gy, k = 4;	To investigate potential benefits of eliminating motion margin through liver tumor treated with conformal therapy
McKenzie et al. 2000	A	No	How should motion margin be combined with other margins around CTV
Van Herk et al. 2003	0.7A	α/β = 1~10	To investigate biologic and physical fractionation effects of random geometric errors and respiration motion with Gaussian blurring of the plan dose
Molinelli et al. 2008	0 5 mm 10 mm	EUD(SF_2 _= 0.5) gEUD(a = -20)	To quantify the potential benefits of CTV-to-PTV margin reduction for SBRT of liver tumor
This study	1A 0.7A 0A	D_ref _= 2 Gy; SF_2 _= 0.3, 0.5,0.7	To investigate adequate margin for different clinical stage of liver tumor treated with fractionation IMRT

## Methods

### Data acquisition

Eight patients with unresectable tumors within the liver were planned with non-gated and gated techniques (Varian RPM system). The details of the RPM system have been described in detail previously [25]. Briefly, a small plastic box with infra-red (IR) reflective markers is placed on the patient. An array of IR LEDs illuminates the box while a camera monitors the displacement of the box due to patient breathing. The respiratory motion for each patient was recorded through the observation of the diaphragm movement under the fluoroscopy with the RPM system installed on a Ximatron simulator (Varian Medical Systems, Palo Alto, USA). The diaphragm's movements during a 100% amplitude window (peak to trough) and another 50% amplitude window (mid-peak to trough) were measured in order to expand the CTV and generate a PTV (planning target volume). The maximum motion extent measurements with the 50% amplitude window were made in order to increase the number of analyzed plans in this study consisting of smaller extents of motion. The trajectories of the movements were also plotted as a motion distribution of f_p_(r) where r is the displacement of the diaphragm relative to the end exhalation position p. A total of 11 CTVs from eight patients (three of the patients have two lesions) were contoured from CT in this study. Each patient was planned using both the full and half amplitudes of motion such that sixteen plans were generated in total.

The study with motion from fluoroscopy assumed that the liver was dragged by the diaphragm along the cranial-caudal direction without any changes in size and shape. CT images with 5 mm thickness used for treatment planning were acquired at end exhalation. Calculations were performed at a 2.5 mm grid size. The objectives of the planning were to minimize the mean dose to the normal tissue with at least 95% of the PTV covered with 50 Gy.

In contrast to the motion study without considering the deformation, a single patient was planned with 4DCT images for comparison. The 4DCT image set was acquired using a GE (General Electric Medical system, Buckinghamshire, UK) Lightspeed 16-slice CT scanner with Varian's RPM system. Images were scanned at 0.5 seconds per revolution and a 4DCT protocol developed by Rietzel et al [26] was followed. The reconstructed CT images had a voxel size of 2.5 mm in the superior-inferior (SI) direction, and 1 mm in the anterior-posterior (AP) and right-left (RL) directions. After the 10 phase CT images were sorted and organ motion was studied in the GE Advantage4D workstation, the 10 phase CT images were exported to Varian Eclipse (version 8.6) workstation. A 4D planning scheme was performed which incorporated the patient's motion model in 3D

### Margin design

**Figure 1:**
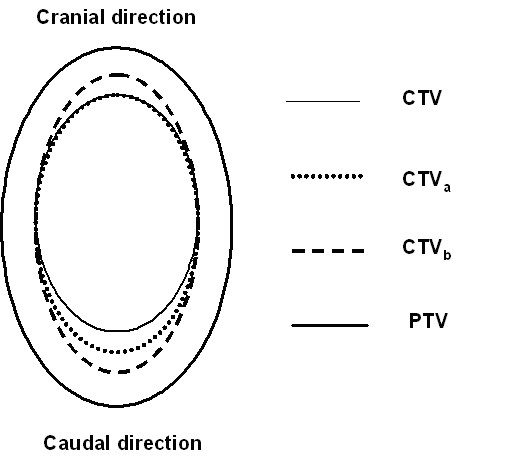
**An illustration of the three margins designed in this study**. 1) CTV applied a full respiratory motion margin in the caudal direction; 2) CTV_a _added a 0.3 A expansion on CTV in the caudal direction to simulate a 0.7 A margin recipe; 3) CTV_b _expanded CTV_a _by 0.5 cm in both cranial-caudal directions to simulate no motion margin recipe.

The planning target volume (PTV) was generated according to recommendations of ICRU Report 62 with internal margins (IM) and setup margins (SM). The CTV to PTV expansion was calculated by the root of the sum of squares of IM and SM. Brock et al [27] have investigated intra-hepatic lesions including an isotropic 5 mm expansion for setup error, an inferior margin for the patient-specific range of the diaphragm movement due to breathing, and an addition 3 mm superior expansion for reproducibility of the exhale state [28]. Based upon the margin design in Brock's definition of PTV, we compared the effect of geometric uncertainty with different margin recipes. Conventionally, we would select a PTV margin around a CTV. The aim of this study was to obtain information on the discrepancy between the static and motion blurred IMRT dose distribution resulting from geometrical error. To reduce the number of plan calculations, we considered the reverse approach, which is to keep the PTV constant, but change the size of the CTV (figure 1). Hence, we introduce the CTV, CTV_a _and CTV_b _described as follows.

1) The lesion on CT images enhanced with contrast was defined as the CTV. The full diaphragmatic motion (the amplitude of respiration) and the PTV expansion method described above were applied for the CTV margin.

2) To comply with Van Herk's 70% motion margin recipe, the original CTVs were expanded 0.3 A caudally as CTV_a _such that CTV_a _could construct the same PTV from an expansion of Van Herk's margin.

3) We approximate elimination of the motion margin by creating CTV_b _with the addition of another 0.5 cm margin to CTV_a _(i.e. one CT slice, due to CT thickness, the exact number of full motion elimination was difficult to reached) in both cranial and caudal directions. This expansion simulates a further margin reduction in the cranial-caudal direction from the PTV.

With the above approximations, all three CTVs are expanded to the same PTV. CTV is the original clinical target volume, CTV_a _is close to a clinical target volume with a reduced margin of 0.7A, and CTV_b _is close to a clinical target volume excluding a motion margin but including a setup margin.

### Incorporation of geometric errors: Two step convolution

Convolution is a mathematical model for combining two functions into a third function. It is an established procedure for converting an input object (function 1, either an image, fluence, or dose distribution) in motion (function 2, either a filter, motion distribution, or probability distribution) into an output object (function 3, same attribute as function 1) with the motion smearing effect. When the input object is an image, motion will blur the image in the same way that a moving object is blurred in photography. When the input object is a fluence or dose distribution, motion will blur the fluence or dose. Convolution incorporates the motion function to output an object which simulates the blurred image, fluence, or dose distribution. Motion smearing of the fluence or dose distribution causes the broadening of the penumbra and the degradation of the target coverage. This study applied a two-step convolution to simulate the dose received by the patient. The first step convolved the fluence created by the MLC with the patient's moving diaphragm distribution to generate an effective fluence. The second step convolved the dose matrix calculated from the effective fluence with a Gaussian distribution representing the setup error. The first step considered the inhomogeneity of the body. The second step assumed that the body is homogeneous. The effective fluence method for the dose distortion by patient motion has been validated in our previous study [15].

Sixteen IMRT plans were created for PTV and PTVg (maximum motion extent taken from the 50% motion window) of eight liver patients for a total of 24 CTVs. The dose prescription was 50 Gy to the PTV with 2 Gy per fraction (25 fractions) for each plan. The dynamic MLC motion files from the planning system (Varian, Cadplan, Varian Medical Systems, Palo Alto, USA) were then convolved with the motion function [11] to obtain the effective fluence(1)

where *x*^*k*^_*r *_*(t) *and *x*^*k*^_*l *_*(t) *denote the positions of the right and left leaves (relative to the iso-center), respectively, of the *k*th leaf pair. *I *is the intensity distribution generated from the leaf motion (which is in perpendicular direction to the diaphragm movement in this study). For an arbitrary point in the organ, p, if the fluence has no distortion due to the motion of an organ, χ is constant. In the calculation of the fluence following distortion due to organ motion, χ is substituted for the pre-known motion function,(2)

Where *f*_*p *_() is the motion function for point p; *ζ *is the period; *A *is the motion amplitude; *t *is the beam-on-time, and *t*_*o *_is the initial phase. In the calculation of the convolution, the specific patient motion trajectory distribution f_p_() acquired in previous section was incorporated into equation (1). During the convolution process, the initial phase was randomly sampled 25 times to simulate 25 different treatments. The static fluences and the effective fluences were incorporated back to the planning system for forward dose calculation [29]. The dose distributions of static fluences were considered as static plans without motion and the dose distributions of effective fluences were considered as plans incorporating motion.

After the dose distribution was obtained from the effective fluence, a second convolution was performed by convolving the dose matrix with a three dimensional Gaussian probability distribution function [30,31],(3)

where D_m _is the dose matrix incorporating with motion effect, D_mr_(R) is the expected dose distribution (at R(x,y,z)) blurred by the random setup uncertainty. The random setup uncertainty is described by an isotropic 3-D Gaussian distribution (PDF_G_) with a standard deviation of 0.5 cm in the anterior-posterior (AP), lateral (LAT), and caudal-cranial (CC) directions [31].

### Systematic Error

Systematic error might occur during the image acquisition or treatment execution. To compare the effects of random and systematic errors, an arbitrary offset of 0.5 cm (in contrast to random error and the criteria of an acceptable tolerance in practice) was applied at isocenter for the effective fluence before the final dose calculations were done at 1) caudal (-z) direction; 2) cranial (+z) direction; 3) 0.35 cm at both of the right lateral (x) and anterior (y) direction. The latter represents the expansion in both x and y directions such that (x^2^+y^2^)^1/2 ^= 0.5.

### Plan Evaluation

EUD as defined by Niemierko [32] is given by,.(4)

Equation 4 was used to compare the effect of geometric uncertainties on EUD by varying the parameter SF_2 _over a range of values (0.3, 0.5, and 0.7) to represent very radiosensitive, medium radiosensitive, and radioresistant tumor cell types, respectively. D_ref_, the reference fraction dose, was 2 Gy in this study in conjunction with SF_2_.

The static plans were compared with plans incorporating geometric uncertainties for CTV, CTV_a_, CTV_b _and PTV in terms of cold spots, as defined by: 1) the dose encompassing 99% of the volume (D_99%V_), 2) the fraction of the target volume with dose 10% (V_90%D_), and 5% lower (V_95%D_) than the prescription dose. To compare the biological effects, DVHs were converted to equivalent uniform dose (EUD), using equation (4), then the impact of geometric uncertainty was calculated as the percentage of dose error:(5)

where EUD_GU_, and EUD_plan _are the EUD with and without geometric uncertainty, respectively.

### Statistical Analysis

The plan evaluation results at the above end points (D_99%V, _V_90%D_, V_95%D_, %(ΔEUD) at SF_2 _= 0.3, 0.5, and 0.7) for different margins (different CTVs) were analyzed using SAS software (SAS institute inc., release 8.1). The statistical significance of the difference between these end points was determined using a two sided paired sample t test, where the end points of the CTVs are paired by patient. Differences of the results were reported to be significant at p < 0.05.

### Comparison with 4D study

A study incorporated 4D CT data which accounted for the organ deformation during respiration was compared with the study above. The details of the 4D method were mentioned in a separate report [33]. In brief, a 4D plan was done by warping the static dose distribution of different phases of CT images with a 3D deformation map such that the overall dose at each tissue voxel was accumulated at the reference CT image. The 3D deformation map was generated after deformable registration registered 4D CT images into the reference CT image. A diffeormorphic registration algorithm was built upon ITK's (Insight Segmentation and Registration Toolkit) environment to perform the deformable registration. Another in-house program was developed to synchronize dynamic MLC segments with respiration phases such that static dose distribution of different phases can be obtained from the sorted synchronized DMLC segments. To account for the random set up uncertainty, a convolution similar to equation (3) was applied at the static dose distribution of each respiration phase before the dose distribution was warped with the deformation map. After the dose warping, the deformed dose distributions from each respiratory phase were summed together as the simulated 4D dose distribution.

## Results

**Figure 2:**
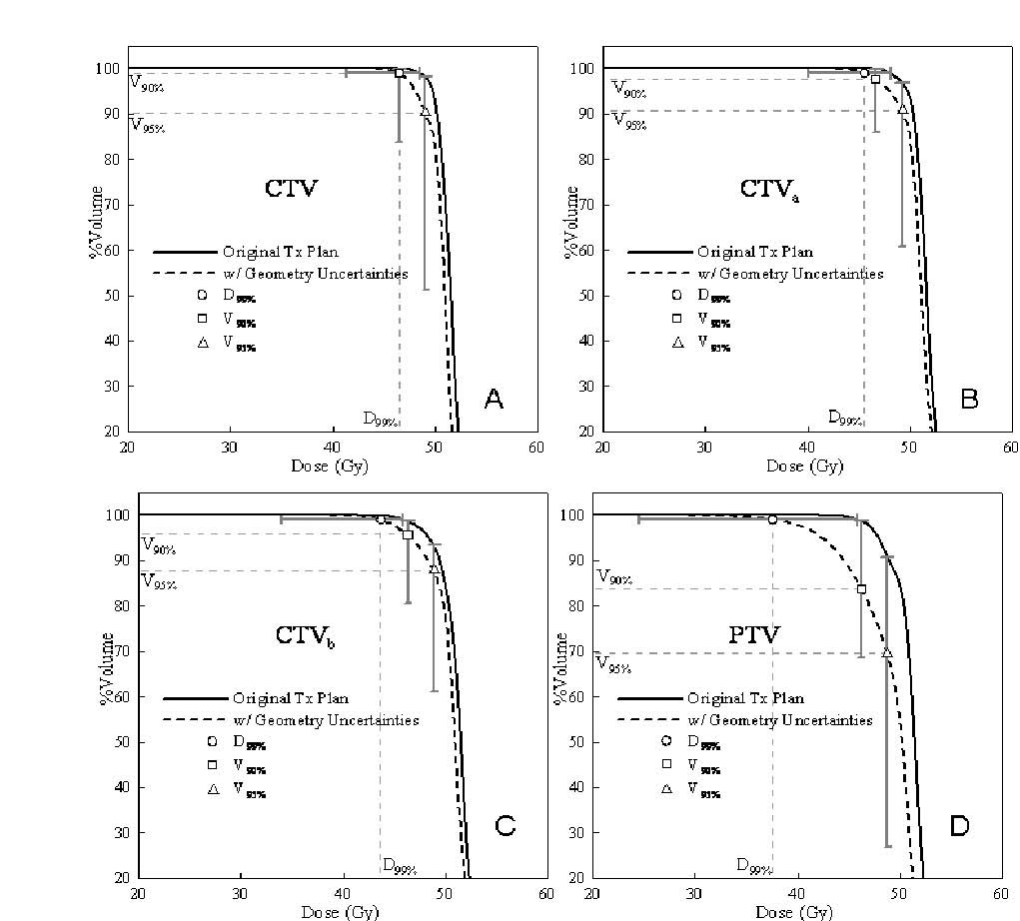
**The effects of geometric uncertainty on the DVH of different targets**. The DVH curves are the mean curves from different patients in this study. (a), (b), (c) & (d) correspond to CTV, CTV_a_, CTV_b_, and PTV, respectively. The ο, □, Δ symbols show the mean reduction of the D_99%V_, V_90%D_, and V_95%D _from the original DVH curves. The bar across the symbols are the range of the data set.

The CTV volumes ranged between 7 and 206 cc (mean 88 cc) and the motion amplitudes ranged from 0.9 to 1.9 cm (mean 1.33 cm). The three different CTVs (CTV, CTV_a _and CTV_b_) and their volumes with PTV margins are listed in table 2. Figure 2 shows dose volume histograms (DVH) averaged over the patient population. The solid lines refer to the original plan and the dashed lines refer to the plan with the effects of geometric uncertainties due to organ motion and random setup error. Also shown are the different data points of intrest (D_99%V, _V_90%D_, and V_95%D_). The error bars on these points indicate the range over the patient population. Since the margin for the CTV and CTV_a _are sufficient to overcome the effect of geometric errors, their DVHs with and without motion are effectively identical. The mean ±1SD of the D_99%V, _V_90%D_, and V_95%D _for CTV after the geometric uncertainties were 5.2 ± 3.9 (Gy), 1.3 ± 3.7 (%), and 7.7 ± 13.6 (%), respectively. Despite a slightly smaller margin than CTV_a_, similar results hold for CTV_b_, although the coverage for the chosen end points, on average, is lower. Specifically, the mean ±1SD of the D_99%V, _V_90%D_, and V_95%D _after the geometric uncertainties were 12.3 ± 6 (Gy), 3.2 ± 4.6 (%), and 8.6 ± 8.6 (%), respectively. Finally, in Figure 2(d), the PTV can be considered as a target without any margin for geometric uncertainties. Here, the effect of motion is dramatic and a large portion of the target volume will receive an unacceptably low dose.

**Table 2:** Patient data with amplitudes, targets and the margins in this study (P3, P5, and P6 have two lesions).

patient	Amplitude	Volume (cc)			
	cm*	CTV	**CTV**_**a**_	**CTV**_**b**_	PTV
P1	1.4	89	121	161	213
	0.7				175

P2	1.7	206	266	344	439
	0.9				352

P3	1.8	89;23	148;44	222;70	291;103
	1				220;86

P4	1.9	90	126	191	367
	1				314

P5	1.2	77;7	107;17	149;31	183;43
	0.6				152;40

P6	0.9	111;42	139;57	184;77	250;117
	0.5				214;111

P7	1.1	163	215	287	325
	1.1	15	28	45	61

P8	1.6	141	200	284	791
	0.8				645

* Each patient was planned with the full and half amplitudes of motion such that sixteen plans were generated in total.

**Figure 3:**
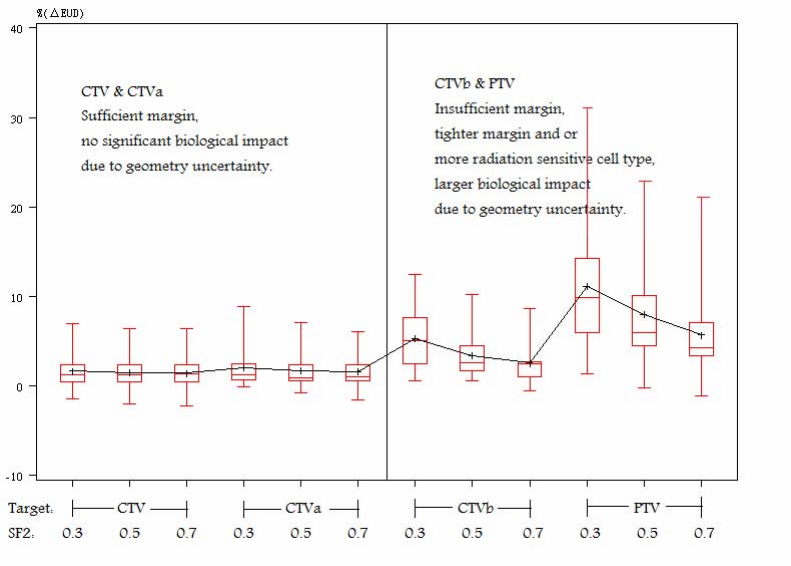
**Box plot of %(ΔEUD) vs. different CTVs with different radiation sensitivity**. The range indicated by the error bars, the 1^st ^and median and the third quartiles, shown as a line, and upper and lower limits of the box, respectively and the average indicated by the points for different margins as indicated by CTV, CTV_a _and CTV_b_.for a) SF_2 _= 0.3, b) SF_2 _= 0.5, and c) SF_2 _= 0.7 on the x-axis.

Figure 3 shows a box plot of the variation of the biological response %(ΔEUD) to the presence of geometric uncertainties for different targets and different survival rates. Both the value and standard deviation of %(ΔEUD) increase as the margin decreases. The box plot also shows how %(ΔEUD) changes as survival rates (SF_2_) change. There is no significant change as SF_2 _increases for both of CTV and CTV_a_. The geometric uncertainties induced biological effects quantified as %(ΔEUD) error, were 1.4 ± 1.9%, 1.4 ± 1.9%, and 1.6 ± 1.9% for SF_2 _of 0.7, 0.5 and 0.3, respectively. For CTV_a_, the values of %(ΔEUD) were 1.6 ± 1.8%, 1.7 ± 2.0%, and 2.1 ± 2.3% for SF_2 _of 0.7, 0.5 and 0.3, respectively, which are statistically indistinguishable from CTV. However, for CTV_b _and PTV, the mean value and variation (as measured by range and standard deviation) both decrease with as SF_2 _increases. The biological effects were 2.5 ± 2%.1, 3.4 ± 2.4%, and 5.3 ± 3.5% for SF_2 _of 0.7, 0.5 and 0.3, respectively, for CTV_b_. The biological impact was largest when there were no motion and setup margins at all (i.e. when CTV = PTV).

To establish if the results from figures 2 &3 are statistically significant, a paired sample t test was used to compare the difference between CTV and CTV_a _and the difference between CTV and CTV_b _in terms of D_99%V, _V_90%D_, V_95%D_, %(ΔEUD) at SF_2 _= 0.3, 0.5, and 0.7. The results of this test are listed in Table 3. The geometric uncertainty has the same effect on physical and biological DVH for CTV and CTV_a_. However, the difference in dosimetric impact between CTV and CTV_b _is significant using most endpoints. We calculated the correlation between the %(ΔEUD_0.5_) on CTV_b_, motion amplitude, the amount of margin on CTV_b_, and the %(ΔEUD_0.5_) on CTV. The correlation coefficients between CTV_b _and the rest of the parameters (amplitude, margin, and %(ΔEUD_0.5_) on CTV_b _were 0.14, 0.58 and 0.32, respectively. The strongest correlation between the %(ΔEUD_0.5_) on CTV_b _is with the margin size (or more specifically, the lack thereof).

**Table 3:** The p-values of the paired sample t test for CTV with CTV_a_, and CTV with CTV_b_.

	**CTV**_**a**_	**CTV**_**b**_
D_99%V_	0.049	<0.0001
V_90%D_	0.1231	0.0066
V_95%D_	0.7049	0.6471
%(ΔEUD_0.7_)	0.4017	0.0002
%(ΔEUD_0.5_)	0.2526	<0.0001
%(ΔEUD_0.3_)	0.088	<0.0001

Two data sets are the statistically the same if the p-value is larger than 0.05.

**Figure 4:**
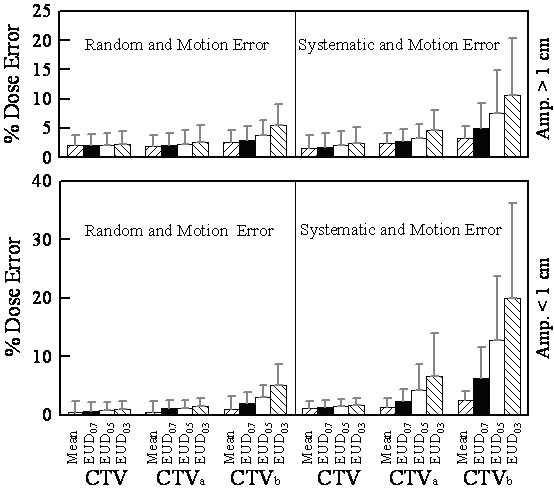
**The effects of motion plus random error and motion plus systematic error**. The percentage dose errors are shown for patients with motion amplitudes greater than 1 cm and less than 1 cm. Data are shown for different margin recipes resulting in different CTVs (CTV, CTV_a_, and CTV_b_) and over a variety of different radiosensitivities (SF_2 _= 0.7, 0.5 and 0.3). The effects of motion plus random error and motion plus systemic error are separated in the two graphs. The error bars indicate 1 standard deviation.

Figure 4 displays the dosimetric error (equation 5) comparisons for both the motion plus random and motion plus systematic errors. We grouped the data into subsets, one for amplitude > 1 cm and the other < 1 cm. The data are shown for different CTVs resulting from the different margin recipes over a range of tumor cell types. The systematic errors (combined with motion error) displayed in the figure were the worst case of the three simulated center offsets (as systematic errors) in the caudal-cranial and the transverse directions. The results showed that as we combined motion and random error, CTV_b _had a mean %(ΔEUD) reduction of 3%~6%, which made no significant difference between the two groups. On the contrary, combined motion with systematic error, for the group of motion amplitude > 1 cm, the CTV_a _had a mean %(ΔEUD) reduction of 2%~5%, CTV_b _had a mean %(ΔEUD) reduction of 3%~11%. These mean %(ΔEUD) errors were twice as high for the group of motion amplitude < 1 cm.

**Figure 5:**
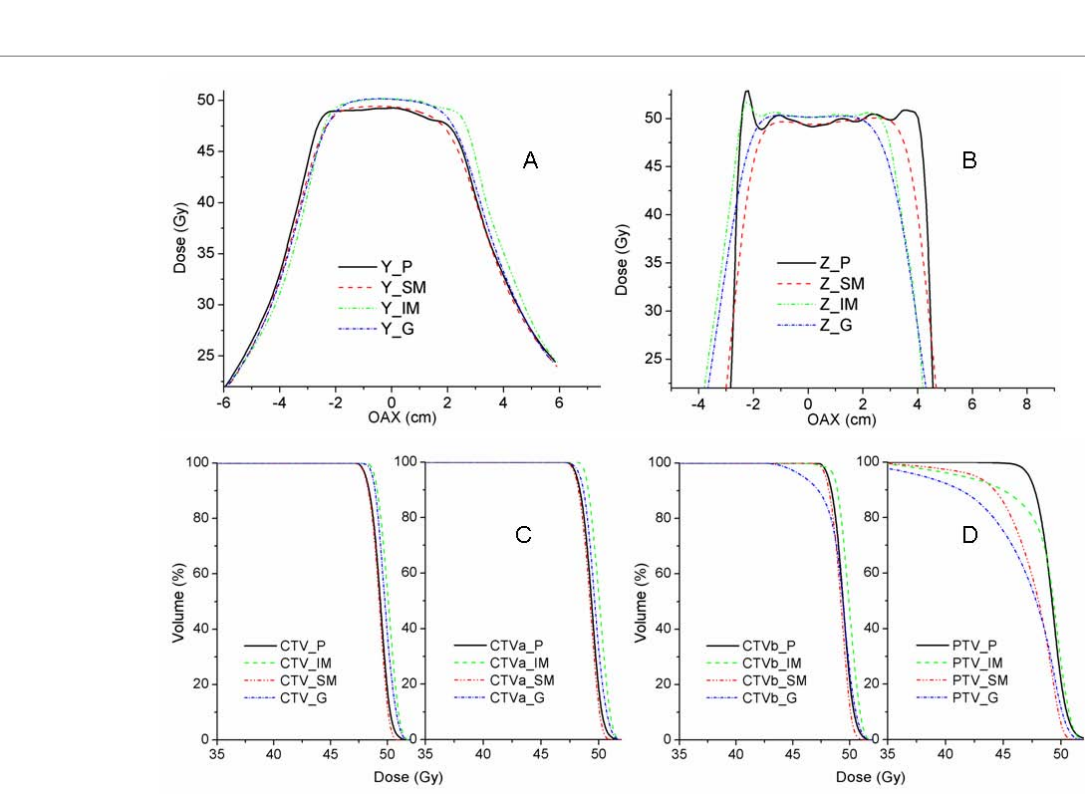
**Effects of geometrical uncertainty on the patient with 4D planning**. (a) & (b) display dose profiles (OAX is the off axis distance) of the static plan ("P") and with different geometric impact s("SM", "IM" &"G") in "Y" and "Z" directions, respectively. (c) & (d) display the DVH of the plans with and without different geometric uncertainties for targets with sufficient margins (CTV & CTV_a_) and without sufficient margins (CTV_b _& PTV).

For the case with 4D data, figure 5a and 5b display the dose profiles without geometry effects ("P"), with set up error ("SM"), with motion error ("IM"), and with geometry impact ("G", combines "SM" & "IM") in AP ("Y"), and Sup/Inf ("Z") directions, respectively. The motion in the RT/LT direction is not shown since the effect is smaller than the effect in the AP direction. The displacements of the 95% dose position from planning iso-center due to different geometry uncertainty are summarized in table 4. The degradations (negative value) of the 95% dose position were 3 mm, 2.4 mm, and 12.9 mm in RT/LT, AP, and Sup/Inf directions, respectively. Set up uncertainty has effect relatively isotropic in all directions. Respiration motion dominates the geometry impact in the inferior direction only. Figure 5c and 5d show the DVH of the plans with and without different geometry uncertainties for targets with sufficient margins (CTV & CTV_a_) and without sufficient margins (CTV_b _& PTV). The EUD errors from the geometry errors are summarized in table 3 for SF_2 _of 0.3, 0.5 and 0.7, respectively. The geometric error impact is insignificant for CTV and CTV_a_. It has a small impact for CTV_b _of sensitive cell type (SF_2 _equal to 0.3).

**Table 4:** The motion characteristics of the case plan with 4D scheme.

	Lt/Rt	AP	Sup/Inf
Max. displacement	1.6 mm	5.0 mm	13.0 mm
95%_SM	-2.5/-3.3	-1.8/-1.6	-4.0/-5.0
95%_IM	+1.0/-0.4	-2.4/+3.0	+1.4/-8.8
95%_G	-1.3/-3.2	-2.4/+2.6	-1.9/-12.6

	EUD_SF0.3_	EUD_SF0.5_	EUD_SF0.7_

CTV_G	1.010	1.010	1.009
CTV_a__G	1.006	1.006	1.006
CTV_b__G	0.976	0.986	0.990
PTV_G	0.788	0.854	0.909

The change of the 95% dose position of the dose profiles due to different geometry uncertainties and biological impact (EUD) for different margins are summarized.

## Discussion

Margin design is critical for the dose received by the tumor. An optimum margin is the aperture that ensures the dose received by target with the least possible amount of irradiation of normal tissue. In this study, three different margin recipes were tested. Both CTV to PTV and CTV_a _to PTV are sufficient to accommodate respiration and setup error. Our T-test results at different end points all indicated that the margin recipe of CTV can be replaced by margin recipe of CTV_a_; however, the margin recipe of CTV_b _cannot replace the margin recipe of CTV without any compensation.

The CTV_b _has insufficient margin by 0.5 cm to 1 cm. For the cases of motion amplitude larger than 1 cm (table 2), the margin from CTV_b _to PTV is close to the margin with random setup error only. This approximates the case in Ten Haken's study [23] where the motion margin was eliminated. In their study, elimination of motion facilitates dose escalation of about 11% for the same normal tissue complication probability. However, due to the dose smearing effect, the geometric uncertainty results in an actual escalated BED by approximately 5~8%, depending upon the radiation sensitivity of the tumor cell. This ignores systematic errors. If systematic errors exist during the treatment, the potential dose escalation would be negated by the geometric delivery inaccuracy. In the group of amplitudes greater than 1 cm in Figure 4 with combined motion and systematic errors, CTV_b _had a mean %(ΔEUD) reduction ranging from 3% to11% and a maximum reduction ranging from 6% to 20%. These also reflect the fact that systematic error has a serious impact on the equivalent dose received by the target compared to random error. The smaller margin the target has (as in the group of amplitude less than one cm), the larger the dosimetric error caused by the 0.5 cm systematic offset. In practice, elimination of the motion margin should be performed with reliable image guidance techniques (IGRT), and a dose escalation scheme should be considered to compensate for the biologic dose reduction due to the geometric misses during treatment.

We caution that our approach is not universally applicable. Here, we reduced the size of the CTV without altering the size of the PTV. In reality, it is the PTV volume that changes. If applied with constant CTV, PTV with bigger margin would have a larger volume. This will lead to different dose uniformity within the PTV and more dose to normal tissue after the inverse planning of IMRT. The dose gradient between the borders of the PTV will be different, too. Since this study compared the coverage of CTV with and without motion impact after large (25) fractionation IMRT delivery, slight dose non-uniformity (after inverse planning) within the PTV should not affect the results of the CTV coverage in this study. Slight different dose gradient will affect more at PTV and less at CTVs after the dose smeared by motion. The irradiation to normal tissue after motion impact is outside the scope of this study, too. These are issues warrant further study.

The radiation sensitivity of the cell type has a strong influence on the sensitivity of the target to margin reduction. This conclusion can be drawn from the results shown in figures 3 and 4. Overall, since the dosimetric effect resulting from geometric uncertainties is larger at the target border, where the dose gradient is greater, the radiosensitive tumor cells suffer more from the geometric uncertainties when the margins were insufficient (e.g., CTV_b _in this study). The low dose volume generated by geometric uncertainties has a larger biological impact on more radiation sensitive tumor types. Low dose volumes may be generated during the optimization process. Although the cold spots were outside the CTV, the biological impact could be magnified after the dose blurring by geometrical uncertainties.

**Figure 6:**
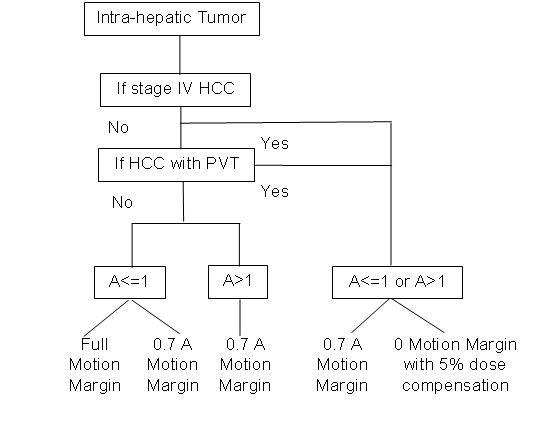
**Clinical implementation of motion margin for intra-hepatic tumor**. PVT is portal vein thrombosis, A is the motion amplitude.

Since the impact of the geometric uncertainties is dependent on the tumor cell type, margin reduction should also consider the clinical stage of the tumor and the corresponding radiation sensitivity. An example of the implementation is shown in figure 6, which is based on the tumor and tumor cell response to radiation at different clinical stages listed at table 5. The survival fractions of stage IIb and IIIb HCC are lower than 0.5, which indicate radiation sensitive cell types, so the planning target should use generous margins (eg. 1^st ^and 2^nd ^margin recipe in this study) to avoid the cold spots from geometric misses. For stage IV HCC or HCC with PVT, the radiation response is poorer and the whole CTV is usually too big to spare the normal liver, so a smaller margin (2^nd ^and 3^rd ^margin recipe in this study) can be considered. If the motion margin is eliminated, 5% dose escalation is suggested to compensate for tumor dose loss from geometric uncertainties as shown in figure 4 where the mean %(ΔEUD) reduction were between 2%~6% for CTV_b_.

**Table 5:** Response dose and survival fraction (SF_2_) for different clinical stage of intra-hepatic tumor.

**SF**_**2 **_**for different clinical stage and pathology cell type (Liu, 2005)**				
		**Clinical stage**		
		
		IIb	IIIb	IV
Pathology typing				
Hepatocellular carcinoma		0.28	0.47	0.61
Bile duct epithelial carcinoma		0.41	0.57	0.78

Radiation dose needed for tumor response				

		response	non response	
		
HCC without PVT (Park, 2002)		50.1 Gy	44.3 Gy	
HCC with PVT (Kim, 2005)		BED 59.9 Gy	BED 55.2 Gy	

This study considers the diaphragm as a surrogate for liver motion and assumed that the liver has rigid motion in Sup/Inf direction only. A case applied 4D planning scheme with 3D deformable organ motion was studied for comparison and is summarized in table 4. In this case, the maximum displacement at RT/LT, AP, and Sup/Inf are 1.6 mm, 5 mm and 13 mm, respectively. The results show that the CTV with sufficient margins (CTV and CTV_a_) have no dosimetric or biological impact from the geometry uncertainty. When there is insufficient margin (6 mm in this case), the EUD errors were 2.4%, 1.6% and 1% for SF_2 _of 0.3, 0.5, and 0.7, respectively. When no margin was applied (PTV), the EUD errors were 21.2%, 15.6% and 9.1%, for SF_2 _of 0.3, 0.5, and 0.7, respectively. Compared with the results of the rigid body assumption in figures 3 & 4, where CTV & CTV_a _have insignificant dosimetric impacts, CTV_b _has mean EUD errors typically between 2~5% (with maximum of 8%), and PTV has mean EUD errors typically between 6~12% ( with maximum 20%). This result demonstrates a similar conclusion by Brock that deformation is insignificant in affecting the dosimetric coverage of the target and the dose received by normal liver [27]. In other words, this comparison validates the expansion of motion margin design (figure 6) in 3D.

This study only considered SF_2 _in the implementation of EUD; in addition, the literature regarding the relationship of the SF_2 _with the cell type of intra-hepatic tumor is very limited. Our method could over simplify the clinical situation. However, the dose dependency of tumor response of different intra-hepatic lesions among different stages of HCC, metastasis lesions, and tumor with or without PVT is very significant. Applying different margin design in clinical practice for a specific patient is not uncommon due to patient's motion amplitude and planning goal of avoiding the RILD of the normal liver. Based on these clinical experiences, this study proposes to combine the laboratory findings, clinical results (compiled as table 5) and the dosimetric effects of geometry uncertainty (summarized as figure 4) in order to design a reasonable and an achievable margin. Of course, further study of the SF_2_, cell type and the dose response of the intra-hepatic tumor are warranted.

## Conclusions

The biological effects of the geometric uncertainties for intrahepatic lesions depend on margin design and intrinsic radiation sensitivity of the tissue. More radiosensitive tumor cells are more sensitive to the margin size. In the simulation of this study, van Herk's 0.7 A margin is feasible if the inter- and intra- reproducibility of the respiratory motion is also considered. Elimination of the motion margin could be beneficial to normal liver sparing with dose escalation, however, the potential dose reduction due to motion blurring on the dose distribution should also be taken into account. The clinical implementation of margin reduction should consider radiosensitivity of the tumor.

## Competing interests

The authors declare that they have no competing interests.

## Authors' contributions

HK designed analyzed and interpreted data. KC participated software programming. WL, CG and SK participated in contouring and helped revise the draft manuscript.

AW, DM, LH and RY participated in study design, data analysis and helped revise the draft manuscript. All authors read and approved the final manuscript.
